# Role of Sodium–Glucose Cotransporter-2 Inhibitors in Managing Polycystic Ovary Syndrome: A Systematic Review

**DOI:** 10.17925/EE.2025.21.1.2

**Published:** 2025-01-28

**Authors:** ABM Kamrul-Hasan, Sunetra Mondal, Fatema Tuz Zahura Aalpona, Lakshmi Nagendra, Deep Dutta

**Affiliations:** 1. Department of Endocrinology, Mymensingh Medical College, Mymensingh, Bangladesh; 2. Department of Endocrinology, Nil Ratan Sircar Medical College, Kolkata, India; 3. Department of Gynecology & Obstetrics, Khaliajuri Upazila Health Complex, Netrokona, Bangladesh; 4. Department of Endocrinology, Jagadguru Sri Shivarathreeshwara Medical College, JSS Academy of Higher Education and Research, Mysore, India; 5. Department of Endocrinology, CEDAR Superspeciality Healthcare, Dwarka, New Delhi, India

**Keywords:** Body weight, dyslipidaemia, hyperandrogenism, metabolic dysregulation, polycystic ovary syndrome, sodium–glucose cotransporter-2 inhibitors

## Abstract

**Background:**

Sodium–glucose cotransporter-2 inhibitors (SGLT2i) can improve metabolic parameters and significantly reduce weight and fat mass. Evidence regarding the use of SGLT2i in polycystic ovary syndrome (PCOS) is limited. The current systematic review compared the efficacy of SGLT2i with placebo or other active comparators in PCOS.

**Methods:**

Randomized controlled trials (RCTs) involving patients with PCOS who are overweight and obese and receiving SGLT2i as intervention and placebo or any non-hormonal comparator as controls were identified through electronic databases. The outcomes of interest included changes in metabolic, hormonal, anthropometric and body composition parameters.

**Results:**

Five RCTs involving 269 participants were included. Canagliflozin, empagliflozin, dapagliflozin and licogliflozin were studied either as monotherapy or in combination with metformin or exenatide. SGLT2i reduced insulin resistance, as evidenced by decreased homeostatic model assessment for insulin resistance and insulin and fasting plasma glucose levels. Reductions in body weight, body mass index, waist circumference and total body fat were observed with most of the SGLT2i. A reduction in dehydroepiandrosterone sulphate (DHEAS) levels was also observed in two RCTs, whereas a decrease in total testosterone level or free-androgen index was not associated with most SGLT2i. Improvements in menstrual irregularity and hirsutism scores were observed. Triglycerides were reduced, while low-density lipoprotein level was slightly increased with SGLT2i in most RCTs. Improvements in body composition and metabolic parameters were most prominent with a combination of SGLT2i with a glucagon-l ike peptide receptor-1 agonist (GLP1RA), while the combination of SGLT2i with metformin showed better effects on hormonal parameters. Adverse effects with SGLT2i were mostly mild and included genital infections.

**Conclusion:**

SGLT2i, when used as monotherapy or combined with metformin or GLP1RA, are a promising therapy for improving metabolic and hormonal parameters in PCOS.

Polycystic ovary syndrome (PCOS) is the most common endocrinopathy affecting women of reproductive age and is characterized by hyperandrogenism, anovulation and insulin resistance (IR).^1^ Women with PCOS have a high risk of developing type 2 diabetes (T2D), dyslipidaemia, hypertension and cardiovascular diseases.^2,3^ IR and hyperandrogenism are closely intertwined in the pathogenesis of PCOS. The current standard of care in PCOS includes combined oestrogen–progesterone tablets as first-l ine agents to target hyperandrogenism.^4^ The use of insulin-sensitizing medications reduces not only IR and metabolic abnormalities but also androgen levels and improves ovulation rates.^5^ Metformin is recommended as the agent of choice for metabolic complications in patients with PCOS. However, its effect on body weight and body composition is minimal. In addition, a meta-analysis showed that metformin failed to reduce fasting blood glucose or insulin levels, indicating the fact that it did not significantly improve insulin sensitivity in women with PCOS who are non-diabetic and overweight.^6^

Sodium–glucose cotransporter-2 (SGLT2) inhibitors (SGLT2i) are anti-diabetic agents that lower blood glucose levels by increasing renal glucose excretion. SGLT2i also cause weight loss, with favourable changes in body composition, and reduce cardiovascular events in patients with and without diabetes.^7,8^ Thus, they appear to be promising agents for patients with PCOS. However, to date, there is limited evidence regarding the use of SGLT2i in PCOS. Two meta-analyses have shown their effectiveness in improving metabolic and hormonal parameters; however, these studies have included heterogeneous trials with different durations and outcomes.^9,10^ The current systematic review aims to evaluate the efficacy of SGLT2i in PCOS compared with placebo or other drugs and to assess their effectiveness in combination with metformin and other agents.

## Methods

### Data sources and search strategy

This systematic review was designed and written following the Preferred Reporting Items for Systematic Reviews and Meta-Analyses (PRISMA) guidelines. It was registered with the International Prospective Register of Systematic Reviews with the registration number CRD42024560088. The search strategy included keywords using connecting words in the format: (“Polycystic ovarian syndrome” OR “Polycystic ovary syndrome” OR “PCOS”) AND (“Sodium-Glucose Linked Cotransporter two inhibitor” OR “Sodium-Glucose Cotransporter-2 inhibitor” OR “SGLT2i” OR “SGLT2I” OR “SGLT2 inhibitors” OR “Empagliflozin” OR “Canagliflozin” OR “Dapagliflozin” OR “Ertugliflozin” OR “Bexagliflozin” OR “Licogliflozin” OR “Sotagliflozin” OR “Luseogliflozin”). A manual search of the reference lists of the full-text articles was also done. A detailed search strategy has been added as Supplemental Digital Content (*Supplementary Material 1*, available in the online version of this article). PubMed and ClinicalTrials. gov were searched for all randomized clinical trials (RCTs) published from any time until March 2024. Only articles in the English language were included.

Four review authors independently screened the titles and abstracts of the retrieved articles for selection based on the set eligibility criteria. Any discord among the reviewers was resolved by either reaching a consensus or consulting with the fifth and sixth review authors. All selected full-text articles were re-assessed to confirm eligibility and data extraction. Wherever required data were missing, the corresponding author(s) were contacted, requesting a full dataset or information about missing data. In duplicate studies or publications, only the most recent and completed study was included in the analysis.

### Study selection

RCTs evaluating any SGLT2i in adult patients with PCOS aged 18–45 years, who are overweight or obese and not receiving hormonal therapy were included in the current review. Studies were selected for inclusion in this review only if the trials compared the changes in hormonal, anthropometric and metabolic parameters before and after treatment with any SGLT2i with those with a placebo or any non-hormonal active comparator used for the same duration.

The diagnosis of PCOS was made according to the 2003 Rotterdam criteria (hyperandrogenism and chronic anovulation with or without polycystic ovarian morphology) or by the National Institutes of Health (NIH) criteria.^11,12^ The intervention involved using any oral SGLT2i as monotherapy or in combination with any non-hormonal medication. The control group(s) consisted of patients treated with a placebo or other insulin-sensitizer medication(s), such as metformin, glucagon-l ike peptide receptor-1 agonist (GLP1RA) or anti-obesity medications alone or in combination.

Studies were excluded if they included patients with diabetes, endocrine disorders that mimic PCOS such as Cushing’s syndrome, non-classic congenital adrenal hyperplasia, ovarian or adrenal androgen-producing neoplasms, exogenous androgen use, uncontrolled thyroid disease or clinically significant abnormal liver function tests; those who are pregnant; those on insulin or insulin secretagogue anti-diabetic agents; those participating in any weight loss programme; those on any form of hormonal therapy within 3 months before screening; or those who received treatment with clomiphene citrate, letrozole or glucocorticoids within 1 month of screening or strong inhibitors or inducers of CYP3A4/5 and CYP3A 7 days before the study treatment. Two animal model trials and two articles having different outcomes of interest were excluded.^13–16^

### Outcomes of interest

The main outcomes of interest were changes in androgen levels, body weight or body mass index (BMI) and IR. Other outcomes studied included changes in body composition parameters, glycated haemoglobin (HbA1c), lipid parameters, blood pressure (BP), assessment of gonadal function using menstrual history and alterations in other hormones such as changes in luteinizing hormone (LH), follicle-stimulating hormone (FSH) and adverse effect (AE) profile.

### Data extraction and quality assessment

Relevant information was obtained from each study included in the analysis, such as the name and registration number of the RCTs, year of publication, name and dose of the SGLT2i used and the comparator drug, sample size, mean age, mean BMI, follow-up duration and the pre-and post-treatment values of the hormonal, anthropometric, and metabolic parameters in both the SGLT2i and the comparator arms. Four authors independently checked the quality of the included trials through the risk of bias (RoB) assessment using the RoB tool in the Review Manager (RevMan) computer program, version 7.2.0.^17^ This tool concentrates on the criteria of sequence generation, allocation concealment, blinding, incomplete outcome data and selective outcome reporting.^18^ Following evaluation, the trials were divided into low, unclear and high ROB. A quantitative meta-analysis could not be done due to the high heterogeneity among the studies.

## Results

### Research results and trial characteristics

**Figure 1:**
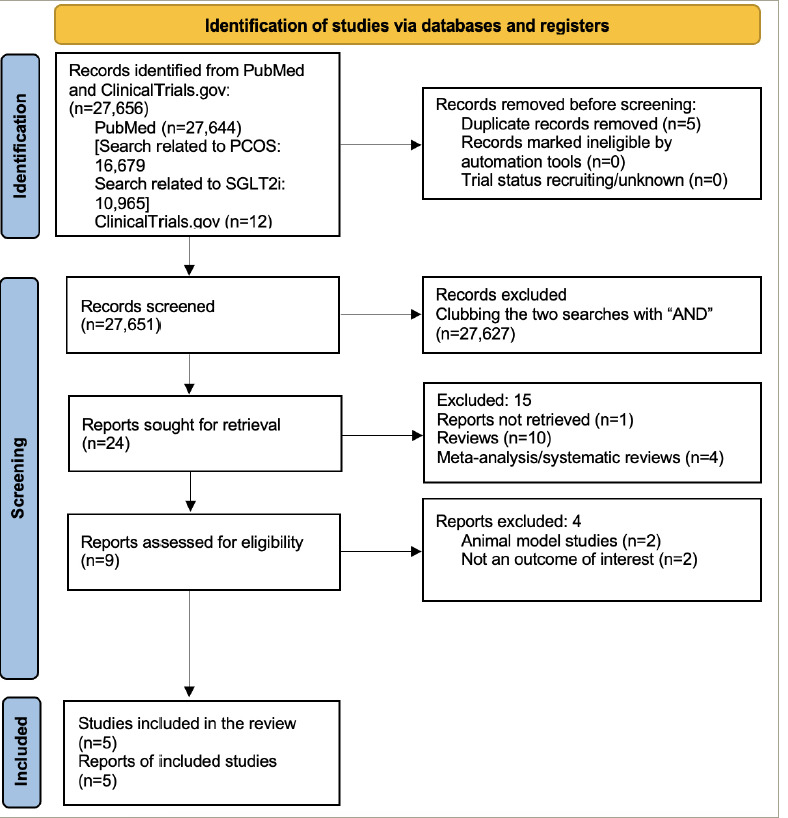
Flowchart on study retrieval and inclusion in the meta-analysis

*Figure 1* presents the flowchart summarizing the literature search from the PubMed database and ClinicalTrials. gov registry. Five RCTs were included in the current study. *Table 1* shows the basic characteristics of the included studies.^11,12,18–20^ In an open-l abel RCT, Cai et al. compared canagliflozin with metformin in women with PCOS.^19^ Elkind-Hirsch et al. compared five treatment arms, namely exenatide weekly, dapagliflozin monotherapy, a combination of these two, a combination of dapagliflozin and metformin and the weight loss medication phentermine–topiramate.^20^ Javed et al. compared empagliflozin with metformin.^11^ Zhang et al. compared the combination of canagliflozin and metformin with metformin monotherapy.^12^ Tan et al. conducted a trial comparing licogliflozin with a placebo.^18^

### Risk of bias in the included studies

**Figure 2:**
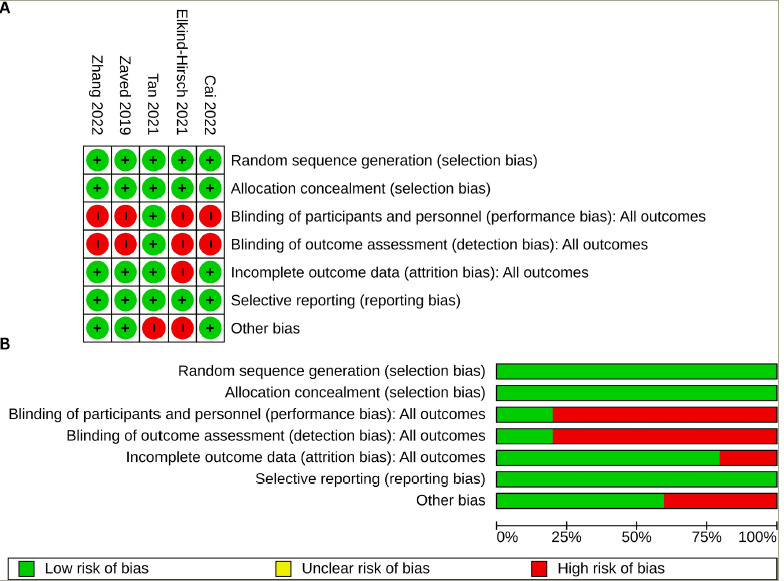
Risk of bias of included randomized controlled trials

The RoB across the five studies included in the systematic review is illustrated in Figure 2. All studies (100%) were assessed as having a low RoB regarding random sequence generation, allocation concealment (selection bias) and selective reporting (reporting bias). The assessment of performance bias (blinding of participants and investigators) and detection bias (blinding of outcome assessors) indicated a high risk for four studies (80%). The risk for attrition bias was low in four studies (80%). Three of the five studies (60%) were considered to have a low risk of other biases. The comprehensive bias risk assessment process is provided as a supplemental file (*Supplementary Table S1*).

*Table 2* summarizes the effects of SGLT2i on the participants’ anthropometric parameters and body composition in the trials.^11,12,19,20^ In the study by Cai et al., significant but similar reductions in body weight, BMI, waist circumference (WC) and hip circumference were observed with both canagliflozin and metformin monotherapy.^19^ In the study by Elkind-Hirsch et al., a combination of exenatide and dapagliflozin (-6 kg and -3.2 kg/m^2^) was more effective in reducing body weight and BMI compared with dapagliflozin monotherapy (-1.4 kg and -0.6 kg/m^2^) or dapagliflozin–metformin combination (-1.8 kg and -1.3 kg/m^2^) (p<0.005).^20^ However, the magnitude of weight loss was highest in the phentermine/ topiramate combination therapy (-9 kg and -3.1 kg/m^2^). Similarly, there was a significantly greater reduction in WC in the exenatide/dapagliflozin arms (-6 cm) compared with dapagliflozin monotherapy (-3 cm) or dapagliflozin/metformin combination therapy (-1.7 cm) (p<0.004). An interesting point was that dapagliflozin/metformin combination therapy led to a lesser degree of weight loss and reduction in WC compared with dapagliflozin monotherapy. In the study by Javed et al., empagliflozin was found to reduce indices of obesity and central obesity, including WC, more significantly than metformin after 12 weeks of treatment.^11^ Zhang et al. found significant reductions in body weight and BMI in both arms.^12^ However, there were no significant differences in body weight between the canagliflozin + metformin versus metformin monotherapy group. In the study by Tan et al., body weight before and after treatment with licogliflozin was unaltered, but the trial duration was only 2 weeks.^18^

*PCOS = polycystic ovary syndrome; SGLT2i = sodium–glucose cotransporter-2 inhibitors.*

### Effects on body composition

In the study by Elkind-Hirsch et al., the combination of exenatide and dapagliflozin (-1.8%) was found to be superior in promoting loss of total body fat percentage (TBF%) compared with dapagliflozin monotherapy (-0.6%).^20^ There was no reduction in TBF% in the dapagliflozin/metformin combination therapy. There was some reduction in the lean body mass in all the treatment arms (p<0.001), the magnitude of which was also highest in the exenatide/dapagliflozin group (-1.2 kg). The exenatide/dapagliflozin combination also significantly reduced the android-to-gynoid ratio (AGR) (-0.06), an effect not observed with the dapagliflozin/metformin combination. Dapagliflozin monotherapy increased mean AGR (0.01). The trunk-to-l eg fat ratio (TLR) was reduced with the exenatide/dapagliflozin combination (-0.13) but increased with dapagliflozin monotherapy (0.01) and dapagliflozin/metformin combination (0.02). Cai et al. reported a decrease in TBF% (-1.58%), subcutaneous adipose tissue mass (-0.2 kg) and visceral adipose tissue mass (-0.08 kg) with canagliflozin.^19^ The loss in TBF% was numerically higher in the metformin arm (-1.58 versus -1.97 for canagliflozin versus metformin, p=0.65). There was no significant reduction in total body lean percentage in either of the groups. Javed et al. reported that empagliflozin significantly reduced total fat percentage (p=0.02), with significant differences in the percentage changes from baseline in fat mass for empagliflozin (-0.7 ± 4.9%) versus metformin (3.2 ± 5.0%) (p=0.023).^11^

**Table 1:** Baseline demographic and basic characteristics of the included randomized controlled trials and participants^11,12,18–20^

				Baseline (mean ± SD)				
Trial details	Major baseline characteristics of the study subjects	Study arms	N	BW (kg)	BMI (kg/m^2^)	HOMA-I R	TT	Study duration
Cai et al. (2022)^19^ NCT04700839 Phase II, single-centre trial from China	Women diagnosed with PCOS by Rotterdam criteria Age 18–45 years Have IR	Canagliflozin 100 mg/day	33	72.9 ± 14.5	27.3 ± 4.8	5.3 ± 3.9	1.8 ± 0.8 ng/mL	12 weeks
		Metformin 1,500–2,000 mg/day	35	73.3 ± 14.4	27.9 ± 5.0	4.6 ± 3.5	1.7 ± 0.8 ng/mL	
Elkind-Hirsch et al. (2021)^20^ NCT02635386 Phase III, single-centre trial in the USA	Women diagnosed with PCOS by NIH criteria Age 18–45 years BMI ≥30 kg/m^2^	Exenatide 2 mg/week	20	105.4 ± 4.0	38.6 ± 1.1	3.9 ± 0.7	47.0 ± 4.2 ng/dL	24 weeks
		Exenatide 2 mg/week + dapagliflozin 10 mg/day	20	104.0 ± 3.0	39.9 ± 0.9	4.3 ± 0.6	47.0 ± 4.3 ng/dL	
		Dapagliflozin 10 mg/day	17	104.0 ± 3.0	38.0 ± 1.1	4.1 ± 0.7	46.0 ± 5.0 ng/dL	
		Dapagliflozin 10 mg/day + metformin 2,000 mg/day	19	103.0 ± 4.0	37.6 ± 1.1	4.6 ± 0.7	45.0 ± 3.5 ng/dL	
		Phenformin/topiramate (7.5/46 mg/day)	16	106.0 ± 4.0	38.4 ± 1.1	3.5 ± 0.8	48.5 ± 4.3 ng/dL	
Javed et al. (2019)^11^ NCT03008551 Phase III, single-centre trial in the UK	Women diagnosed with PCOS by Rotterdam criteria Age 18–45 years BMI ≥25 kg/m^2^	Empagliflozin 25 mg/day	19	102.3 ± 16.6	37.1 ± 6.2	2.6 ± 2.1	1.6 ± 0.4 nmol/L	12 weeks
		Metformin 1,500 mg/day	20	108.8 ± 25.3	38.7 ± 7.8	3.7 ± 2.4	1.7 ± 1.2 nmol/L	
Zhang et al. (2022)^12^ NCT04973891 Phase II single-centre trial in China	Women diagnosed with PCOS by Rotterdam criteria Age 18–40 years BMI ≥24 kg/m^2^	Canagliflozin 100 mg + metformin 2,000 mg/day	21	81.2 ± 9.8	31.1 ± 3.0	5.7 ± 5.1	0.9 ± 0.3 ng/mL	12 weeks
		Metformin 2,000 mg/day	20	74.8 ± 8.9	29.3 ± 3.2	4.25 ± 3.39	0.9 ± 0.4 ng/mL	
Tan et al. (2021)^18^ NCT03152591 Phase II, multicentre trial in Germany and the USA	Women diagnosed with PCOS by Rotterdam criteria Age 18–40 years Have IR and BMI ≥27 kg/m^2^	Licogliflozin 50 t.i.d.	15	103.9 ± 14.5	36.8 ± 4.4	7.54 ± 4.61	1.9 ± 0.8 nmol/L	2 weeks
		Placebo t.i.d.	14	106.6 ± 22.8	39.5 ± 7.7	6.2 ± 3.3	2.1 ± 0.6 nmol/L	

*BMI = body mass index; BW = body weight; HOMA-IR = homeostatic model assessment for insulin resistance; IR = insulin resistance; NIH = National Institutes of Health; PCOS = polycystic ovary syndrome; SD = standard deviation; t.i.d. = three times in a day; TT = total testosterone.*

### Effects on glycaemic parameters and insulin indices

The effects of SGLT2i versus control groups on the glycaemic parameters and insulin indices are summarized in *Table 3*.^11,12,19,20^ Cai et al. found a reduction in homeostatic model assessment for insulin resistance (HOMA-I R) at 12 weeks with both canagliflozin and metformin, canagliflozin being non-i nferior to metformin (treatment difference least square mean [LSM] -0.85 [-2.19 to 0.49]) in the per-protocol analysis (margin for non-i nferiority being 0.6).^19^ A reduction in fasting insulin levels and fasting plasma glucose (FPG) and post-prandial glucose values were also similar between the two (*Table 2*). In the study by Elkind-Hirsch et al., reductions in the HOMA-IR, FPG, glucose excursion during an oral glucose tolerance test (OGTT) and mean plasma glucose (MPG) were observed in all treatment groups (p≤0.0001) after 24 weeks.^20^ Patients receiving exenatide/dapagliflozin combination had significantly greater reductions in MPG levels (-18 mg/dL) compared with dapagliflozin (-3.6 mg/dL) and dapagliflozin/metformin combination therapy (-2 mg/dL) (p<0 .02). None of the participants in any of the treatment arms progressed to T2D. In the study by Javed et al., no significant changes were observed in markers of insulin sensitivity (insulin, FPG and HOMA-I R) with either empagliflozin or metformin, nor were there any differences between the groups.^11^ In the study by Zhang et al., while both the groups demonstrated reductions in fasting insulin levels and HOMA-I R, significant decrease in FPG (-0.33 [-0.95 to -0.05]), area under the curve for glucose (AUCGlu; -158 ± 225.4 mmol/L min) and area under the curve for insulin (AUCIns; -4,264 ± 5,627 mU/L min) levels were observed only in the group receiving canagliflozin/metformin combination (p<0.001 for all).^12^

Data on insulin sensitivity indices (ISIs) were available for two studies. Cai et al. reported improvement in the ISI, HOMA-I SI, with both canagliflozin (0.42 [0.23–0.62]) and metformin (0.29 [0.08–0.50]) to a similar extent (p=0.38).^19^ Elkind-Hirsch et al. found OGTT-derived ISIs to improve significantly after 24 weeks with all drug therapies.^20^ The insulinogenic index (IGI) corrected for the relative level of IR (IGI/HOMA-I R) was significantly improved with the dapagliflozin–exenatide combination (+0.31) or exenatide monotherapy (0.33) but not in the dapagliflozin monotherapy (0) or the dapagliflozin–metformin combination group (-0.1) (p<0.001). On a similar note, the insulin secretion–sensitivity index (IS–SI) improved significantly in the exenatide/dapagliflozin combination (198) and the exenatide monotherapy arm (155) to a much greater extent than that with dapagliflozin monotherapy (33) or dapagliflozin/metformin combination (5) (p<0.03), indicative of better beta-cell functioning in the former groups. Tan et al. demonstrated that licogliflozin reduced hyperinsulinaemia (AUCIns) by 68%, insulin peak by 74%, HOMA-I R by 30% and FPG by 6%.^18^

*(A) Risk of bias summary: review authors’ judgements about each risk of bias item for each included study; (B) risk of bias graph: review authors’ judgements about each risk of bias item presented as percentages across all included studies*

### Effects on lipid parameters

The effects of SGLT2i versus control groups on lipid parameters are summarized in *Table 3*. In the study by Cai et al., both canagliflozin and metformin groups demonstrated similar decreases in serum triglycerides.^19^ There was a slight increase in low-density lipoprotein cholesterol (LDL-C) levels with canagliflozin use (0.19 mmol/L [-0.24 to 0.63]) (*Table 4*).^11,12,18–20^ In the study by Elkind-Hirsch et al., LDL-C levels were reduced in the exenatide–dapagliflozin combination arm (-6 mg/dL) but increased with dapagliflozin alone (6.5 mg/dL) or dapagliflozin/metformin combination (3 mg/dL) (p=not significant).^20^ Zhang et al. found a significant reduction from baseline in triglycerides (-0.27 ± 0.51 mmol/L) and total cholesterol (-0.22 ± 0.4 3 mmol/L) only in the canagliflozin/metformin group (p<0.005).^12^

### Effects on other cardiovascular disease risk factors

In the study by Elkind-Hirsch et al., both systolic blood pressure (SBP) and diastolic blood pressure (DBP) were reduced significantly in all the treatment arms (p<0.035), although no significant between-group differences were observed.^20^ Numerically, the highest reduction in SBP was observed with exenatide monotherapy (-6 mm Hg) and in DBP with exenatide/dapagliflozin combination (5 mm Hg). Javed et al. failed to demonstrate any significant reduction in SBP, DBP, markers of endothelial dysfunction such as augmentation index or radial hyperaemic index or high-sensitivity C-reactive protein with either empagliflozin or metformin arms.^11^

### Effects on hirsutism, menstrual cycle and hormonal parameters

The effects of SGLT2i versus control groups on hirsutism, menstrual cycle and hormonal parameters are summarized in *Table 4*. Cai et al. observed a significant reduction in the levels of dehydroepiandrosterone sulphate (DHEAS) in those receiving canagliflozin (LSM change: 68.96 μg/dL for canagliflozin versus 52 for metformin, p=0.01). However, no differences were noted in the levels of other androgens, including total testosterone (TT), free testosterone, androstenedione and sex hormone-binding globulin (SHBG) levels, or LH and FSH, between the two groups. In addition, the Ferriman–Gallwey (FG) score was similar in the two groups at all points from baseline to the end of the follow-up (*Table 3*).^19^ In their five-arm trial, Elkind-Hirsch et al. found a significant improvement in the levels of TT and free-androgen index (FAI) in all the treatment groups, with the highest reductions in TT levels and FAI observed with dapagliflozin monotherapy (-0.38 nmol/L and -2% for TT and FAI, respectively), followed by exenatide monotherapy (-0.28 nmol/L and -1.5%), both of which were higher than the combination of dapagliflozin/exenatide (-0.22 nmol/L and -1.5%), and the least reduction was observed with dapagliflozin/metformin (-0.19 nmol/L and -0.7%) (p<0.001).^20^ There was an increase in SHBG levels and a non-significant but similar reduction in DHEAS in all groups. The effects on hirsutism or menstrual cyclicity were not studied. Notably, four participants became pregnant during the trial, which indicates the return of ovulatory function. In the study by Javed et al., empagliflozin use led to significant increases in SHBG and oestradiol levels.^11^ However, there were no substantial changes in TT, FAI or DHEAS and no between-group differences. The magnitude of change in the hormone levels from baseline was not different between the empagliflozin and metformin arms. Zhang et al. reported similar improvements in menstrual irregularities with both canagliflozin/metformin combination (80.95%) and metformin monotherapy (80.00%).^12^ A reduction in TT levels was significantly higher in the canagliflozin/metformin group compared with metformin alone. The FAI% was found to decrease significantly only in the canagliflozin/metformin combination group (19.15 ± 13.19 versus 28.62 ± 16.4, p<0.005), while SHBG increase was observed only in the metformin monotherapy group at 12 weeks (*Table 2*). Tan et al. demonstrated that a reduction in DHEAS levels with licogliflozin was significantly higher than placebo (effect size of 24%, treatment ratio 0.76, 90% CI: 0.65–0.89, p=0.008) and numerically greater reductions in TT (9%) and FAI (21%) with licogliflozin.^18^

**Table 2:** Effects of sodium–glucose cotransporter-2 inhibitors compared with other agents on anthropometric and body composition parameters in polycystic ovary syndrome^11,12,19,20^

		Change in anthropometric indices				Changes in body composition
Study	Study medication	Weight (kg)	BMI (kg/m^2^)	Waist circumference (cm)	Waist–hip ratio	Body fat (%)
Cai et al. (2021)^19^	Canagliflozin	-2.82 (-3.97 to -1.66)	-1.04 (-1.56 to -0.53)	-4.05 (-6.18 to -1.91)	-0.02 (-0.04 to 0.00)	-1.58 (-2.63 to -0.53)
	Metformin	-2.68 (-3.93 to -1.43)	-0.90 (-1.46 to -0.35)	-3.27 (-5.54 to -0.99)	-0.01 (-0.03 to 0.01)	-1.97 (-3.34 to -0.60)
	p	0.88	0.73	0.63	0.51	0.65
Elkind-Hirsch et al. (2021)^20^	Exenatide	-4.10	-1.30	-2.0	-0.05	-0.80
	Dapagliflozin	-1.40	-0.60	-3.0	-0.02	-0.60
	Exenatide + dapagliflozin	-6.0	-3.20	-6.0	-0.01	-1.80
	Dapagliflozin + metformin	-1.80	-0.60	-1.70	0	0
	Phentermine + topiramate	-9.0	-3.10	-7.0	-0.01	-2.2
	p	<0.005	<0.005	<0.035	ns	<0.008
Javed et al. (2019)^11^	Empagliflozin	-1.40 ± 3.20	-1.40 ± 3.20	-1.60 ± 2.80	-	0.60 ± 3.20
	Metformin	1.20 ± 2.30	1.10 ± 2.20	0.20 ± 2.10	-	1.10 ± 3.80
	p	0.006	0.007	0.029	-	ns
Zhang et al. (2022)^12^	Canagliflozin + metformin	-6.66 ± 4.24	-2.49 ± 1.55	-	-	-
	Metformin	-5.85 ± 3.32	-2.20 ± 1.30	-	-	
	p	0.54	0.54	-	-	

*BMI = body mass index; ns = not significant.*

### Adverse events

Cai et al. found similar rates of AEs during treatment with canagliflozin (15.15%) and metformin (50.55%), with higher incidences of *pruritus vulvae* and osmotic diuresis-related AEs with canagliflozin compared with metformin.^19^ Patients affected by *pruritus vulvae* mostly had mild symptoms that resolved within a few days of discontinuation. In the trial by Elkind-H irsch et al., urogenital infections were common with dapagliflozin monotherapy. The combination of dapagliflozin with metformin or exenatide did not alter the incidence of infections.^20^ Zhang et al. reported AEs in 57.70 and 68.00%, respectively, in the canagliflozin/metformin and metformin monotherapy groups.^12^

## Discussion

The current systematic review was conducted to evaluate the performance of SGLT2i as a promising alternative to metformin in patients with PCOS who are not on hormonal combined oral contraceptive pills. On analysing the results of the trials, we found that a reduction in HOMA-I R, insulin levels and glycaemic parameters was similar with canagliflozin and metformin, with slightly additional benefits observed when both were combined.^12,19^ In the study with empagliflozin, neither empagliflozin nor metformin improved IR. ISIs were improved with canagliflozin to a similar extent as with metformin. At the same time, in the trial by Elkind-Hirsch et al., the combination of dapagliflozin with exenatide showed better improvement in OGTT-derived ISIs, as well as indices of beta-cell functioning, than dapagliflozin alone.^20^ The least benefit was observed with the dapagliflozin–metformin combination. The dual SGLT1/2 inhibitor licogliflozin improved IR within 2 weeks, although no changes in body weight indicated weight-i ndependent effects on insulin sensitivity.^18^ Metformin corrects dysglycaemia by reducing hepatic gluconeogenesis and lipogenesis and enhancing insulin sensitivity in the peripheral tissues with minimal effects on body weight.^21^

**Table 3:** Effects of sodium–glucose cotransporter-2 inhibitors compared with other agents on glycaemic parameters, insulin indices and lipid parameters^11,12,19,20^

		Changes in glycaemic parameters and insulin indices					Changes in lipid parameters			
Study	Study medication	FPG (mmol/L)	HbA1c (%)	HOMA-I R	HOMA-ISI	Fasting insulin (mU/L)	TC (mmol/L)	LDL-C (mmol/L)	HDL-C (mmol/L)	TG (mmol/L)
Cai et al. (2022)^19^	Canagliflozin	-0.23 (-0.40 to -0.06)	-0.26 (-0.43 to -0.09)	-2.05 (-2.92 to -1.18)	0.42 (0.23 to 0.62)	-7.70 (-11.46 to -3.94)	-0.00 (-0.23 to 0.24)	-0.03 (0.28 to 0.34)	0.13 (-0.04 to 0.30)	0.23 (0.44 to 0.03)
	Metformin	-0.23 (-0.41 to -0.05)	-0.08 (-0.27 to 0.11)	-1.23 (-2.15 to 0.30)	0.30 (0.10 to 0.50	-3.97 (-7.97 to 0.03)	0.17 (0. 17 to 0.50)	0.19 (-0.24 to 0.63	-0.15 (0.39 to 0.09)	-0.12 (0.41 to 0.17)
	p	0.99	0.2	-	0.38	0.2	0.33	0.38	0.21	0.39
Elkind-Hirsch et al. (2021)^20^	Exenatide	-0.18	-	-0.40	Matsuda: 0.40 SI OGTT: 0.40 IS–SI: 155.00	-	-0.08	-0.05	-0.04	0.15
	Dapagliflozin	-0.28	-	-0.70	Matsuda: 0.40 SI OGTT: 0.40 IS–SI: 33.00	-	-0.08	0.17	-0.03	0.20
	Exenatide + dapagliflozin	-0.36	-	-1.70	Matsuda: 1.10 SI OGTT: 1.10 IS–SI: 198.00	-	-0.18	-0.16	-0.03	0.80
	Dapagliflozin + metformin	-0.22	-	-1.30	Matsuda: 0.90 SI OGTT: 0.90 IS–SI: 5.00	-	0	0.08	-0.05	0.20
	Phentermine + topiramate	-0.11	-	-0.30	Matsuda: 1.00 SI OGTT: 1.00 IS–SI: 4.00	-	-0.03	-0.03	-0.03	0
	p	<0.0001	-	<0.04	<0.0001	-	ns	ns	ns	ns
Javed et al. (2019)^11^	Empagliflozin	-0.80 ± 5.80	-	-20.50 ± 84.60	-	-	-1.60 ± 13.7	2.70 (30.20)	-0.60 ± 9.20	-6.70 (35.8)
	Metformin	-2.30 ± 8.00	-	-18.90 ± 53.50	-	-	-2.20 ± 8.50	-3.40 (9.60)	-3.40 ± 9.60	-9.00 (49.8)
	p	ns	-	ns	-	-	ns	ns	ns	ns
Zhang et al. (2022)^12^	Canagliflozin + metformin	-0.33 (-0.95 to -0.05)	-	-1.83 (-3.01 to -0.96)	-	-7.00 (-10.40 to -2.00)	-0.22 ± 0.43	-0.12 ± 0.49	-	-0.27 ± 0.51
	Metformin	-0.11 (-0. 49 to 0.1)	-	-1.29 (-2.90 to -0.05)	-	-4.20 (-9.80 to -0.70)	-0.27 ± 0.48	-0.19 ± 0.50	-	-0.05 ± 0.59
	p	0.2	-	0.4	-	0.46	0.80	0.69	-	0.2

*FPG = fasting plasma glucose; HbA1c = glycated haemoglobin; HDL-C = high-density lipoprotein cholesterol; HOMA-IR = homeostatic model assessment for insulin resistance; HOMA-ISI = homeostatic model assessment for insulin sensitivity index; IS–SI = insulin secretion-sensitivity index; LDL-C = low-density lipoprotein cholesterol; ns = not significant; SI OGTT = insulin sensitivity from sparsely sampled oral glucose tolerance test; TC = total cholesterol; TG = triglyceride.*

SGLT2i reduces glucose via an insulin-i ndependent mechanism by inducing glucosuria and improving insulin sensitivity, predominantly by reducing adiposity through enhancing lipolysis, fatty acid oxidation and adipose tissue browning.^22^ Reducing glucotoxicity and lipotoxicity can improve beta-cell function to some extent. IR is a chief driver in the pathogenesis of PCOS, and SGLT2i, especially canagliflozin and dapagliflozin, are as effective as metformin in reducing IR. However, combining SGLT2i with metformin does not confer any additional benefit.

Obesity treatment is an essential component for PCOS management, with as little as 5–7% reduction in body weight, leading to improvements in IR and hyperandrogenaemia.^23^ Prior studies in patients with PCOS have shown that metformin does not consistently reduce abdominal adiposity, and weight loss is often minimal.^24^ SGLT2i can reduce body weight by multiple mechanisms in both patients with diabetes and individuals who are non-diabetic, overweight and obese, with minimal side effects.^25–27^ They also reduce indices of central obesity, such as WC and subcutaneous and visceral fat.^28,29^ Results of the trials mentioned in this review suggest that empagliflozin, but not canagliflozin monotherapy, reduced obesity indices and central obesity more than metformin. Reductions in obesity and central obesity indices were higher with dapagliflozin monotherapy than with dapagliflozin–metformin combination, with the greatest improvements observed in the dapagliflozin–exenatide combination. SGLT2i induce weight loss by increasing glucosuria and urinary caloric loss, while a GLP1RA suppresses appetite and caloric intake.^30,31^ The complementary mechanisms of action of SGLT2i and GLP1RA in weight loss and glycaemic reduction may represent a novel approach to treating individuals with PCOS who have obesity and/or prediabetes. The lack of weight loss in the licogliflozin trial was likely due to the extremely short duration of the study.

There is now considerable evidence that the cardiometabolic complications of obesity are related to body fat distribution, especially truncal fat and an increase in the android fat distribution.^32,33^ Empagliflozin was better than metformin in reducing total and visceral fat, while canagliflozin was not. Dapagliflozin–exenatide combination, and to a lesser extent, dapagliflozin monotherapy, led to loss of total body fat. However, dapagliflozin monotherapy led to a slight increase in AGR and TLR. As previously reported, TLR and AGR were significantly reduced with the dapagliflozin–exenatide combination, indicative of GLP1RA-mediated central fat redistribution. However, loss of lean mass was also higher with the use of the dapagliflozin–exenatide combination. PCOS is an established risk factor for atherosclerotic cardiovascular disease and other metabolic complications, and a reduction in central and visceral obesity can potentially manifest as alleviation in the cardiometabolic risk and mortality in PCOS.

**Table 4:** Effects of sodium–glucose cotransporter-2 inhibitors compared with other agents on clinical and hormonal parameters in polycystic ovary syndrome^11,12,18–20^

		Effects on androgen levels					Improvement in clinical parameters			
Study	Study medication	TT (nmol/L)	SHBG (nmol/L)	DHEAS (μmol/L)	FT (pmol/L) or FAI	Androstenedione (nmol/L)	mFG score	Improvement in menstrual regularity	LH (IU/L)	FSH (IU/L)
Cai et al. (2022)^19^	Canagliflozin	-0.52 (-1.32 to 0.28)	-4.82 (-19.40 to 9.75)	-1.88 (-3.42 to -0.31)	1.04 (-1.04 to 3.09)	-	-0.26 (-0.68 to 0.15)	1.34 (0.66 to 2.02)	0.53 (-2.75 to 3.80)	-0.09 (-1.13 to 0.94)
	Metformin	0.00 (-0.87 to 0.83)	-13.58 (-31.21 to 4.05)	-0.99 (-0.44 to 2.42)	1.04 (-1.53 to 3.61)	-	-0.20 (-0.67 to 0.26)	1.37 (0.63 to 2.11)	-1.84 (-5.41 to 1.72)	-0.12 (-1.23 to 0.98)
	p	0.41	0.47	0.01	0.99	-	0.84	0.95	0.35	0.97
Elkind-Hirsch et al. (2021)^20^	Exenatide	-0.28	-	-0.24	FAI: -1.50	-	-	-	-	-
	Dapagliflozin	-0.38	-	-0.62	-2.00	-	-	-	-	-
	Exenatide + dapagliflozin	-0.22	-	-0.33	-1.50	-	-	-	-	-
	Dapagliflozin + metformin	-0.19	-	-0.19	-0.70	-	-	-	-	-
	Phentermine + topiramate	-0.1	-	0.03	-0.80	-	-	-	-	-
	p	<0.001	-	ns	<0.001	-	-	-	-	-
Javed et al. (2019)^11^	Empagliflozin	2.60 ± 37	9.90 ± 22.60	1.00 ± 20.1	FAI (%): -7.00 ± 31.40	-2.20 ± 24.40	-	-	-	-
	Metformin	-14.00 ± 33.60	6.40 ± 25.50	8.10 ± 15.00	FAI (%): -9.70 ± 34.00	5.60 ± 59.80	-	-	-	-
	p	ns	ns	ns	ns	ns	-	-	-	-
Zhang et al. (2022)^12^	Canagliflozin + metformin	-1.14 ± 0.79	0.10 (-3.45 to 5.30)	-	FAI (%): -9.47 ± 11.65	-1.26 ± 4.08	-	80.95%	-1.91 (-7.40 to 2.49)	-0.75 ± 2.51
	Metformin	-0.62 ± 0.62	2.95 (-2.15 to 10.30)	-	FAI (%): -5.11 ± 7.40	-1.36 ± 5.52	-	80.00%	0.42 (-7.10 to 4.19)	-0.68 ± 2.17
	p	0.02	0.46	-	0.16	0.96	-	0.62	0.19	0.93
Tan et al. (2021)^18^	Licogliflozin versus placebo	Effect size: 9% ↓ in TT (0.91; 90% CI: 0.77–1.07)	Effect size: 15% ↑ in SHBG (1.15; 90% CI: 0.97–1.36)	Effect size: 24% ↓ in DHEAS (0.76; 90% CI: 0.65–0.89)	Effect size: 12% ↓ in FT (0.88; 90% CI: 0.70–1.11)	Effect size: 19% ↓ in androstenedione (0.81; 90% CI: 0.68–0.99)	-	-	-	-
	p	0.34	0.17	0.01	0.35	0.09	-	-	-	-

*CI = confidence interval; DHEAS = dehydroepiandrosterone sulphate; FAI = free-androgen index; FSH = follicle-stimulating hormone; FT = free testosterone; LH = luteinizing hormone; mFG = modified Ferriman– Gallwey score; ns = not significant; SHBG = sex hormone-binding globulin; TT = total testosterone.*

Reductions in total and free testosterone levels were observed with licogliflozin and dapagliflozin monotherapy, as well as with dapagliflozin combined with exenatide or metformin and with canagliflozin/metformin combination. Canagliflozin and empagliflozin were not found to be more effective than metformin alone in reducing hyperandrogenism, although combined canagliflozin and metformin therapy was. Notably, the reduction in testosterone was higher when used dapagliflozin alone as compared with its combination with metformin or GLP1RA. This was despite less weight loss and IR improvement with dapagliflozin monotherapy. While all of these might be a random finding in a single small study, these point to the need for further studies to elucidate the direct effects of SGLT2i on androgen production at the ovary and androgens.

Significant reductions in DHEAS levels were observed with canagliflozin monotherapy, and licogliflozin reduced DHEAS and androstenedione levels within 2 weeks of therapy. Approximately 40–60% of patients with PCOS are known to have adrenal hyperandrogenism with increased DHEAS.^34^ Many women with PCOS have increased serine phosphorylation and decreased tyrosine phosphorylation in insulin receptors and the CYP17A1 gene, leading to the enzyme’s enhanced 17,20-l yase but not 17α-hydroxylase activity, culminating in increased dehydroepiandrosterone (DHEA) production.^35^ The exact mechanism by which SGLT2i could reduce DHEAS levels is yet unknown. The fact that a reduction in DHEAS was observed only with medications that inhibited both SGLT2 and SGLT1, such as canagliflozin or licogliflozin, could hint at the possibility of a role of SGLT1 in the production of DHEAS. The reduction in DHEAS has been proposed to be one factor to explain the hypouricaemic effect of canagliflozin, as DHEAS-mediated mineralocorticoid receptor activation causes increased sodium reabsorption and reduced water excretion, thereby decreasing uricosuria.^36,37^ However, given that DHEAS is also known to have anti-atherosclerotic roles and anti-obesity effects, much remains to be elucidated regarding this mechanism.

Despite the reduction in androgen levels, data on the effects on hirsutism scores were unavailable for most studies. Apart from the fact that the duration of all the trials was shorter than one hair growth cycle, possibly, the degree of androgen reduction required for a noticeable change in hirsutism was also not achieved with any of the therapies. Neither canagliflozin nor metformin was shown to improve hirsutism in the study by Cai et al.^19^ There was an improvement in menstrual regularity with canagliflozin alone or with metformin, although the improvement was not better than with metformin monotherapy.^12^

The management of PCOS entails not only addressing metabolic and hormonal problems but also alleviating long-term cardiovascular risk. We found that triglycerides were lowered, but LDL-C was increased with SGLT2i in all the trials, although the combination of dapagliflozin with exenatide reduced LDL-C. Similarly, a reduction in DBP was also observed with the exenatide–dapagliflozin combination. There was no significant effect on markers of endothelial dysfunction. Although long-term cardiovascular events were not reported, the reductions in LDL-C, triglycerides and BP, as well as better control of obesity and IR, could potentially result in reduced incidence of atherosclerotic cardiovascular diseases in the long run.^8^

AEs were mostly mild and were predominantly gastrointestinal with metformin and genital infections with SGLT2i. Combining SGLT2i with metformin and/or GLP1RA did not alter the rates or severity of AEs.

## Limitations

Limitations of this systematic review include the inclusion of only a few, small, single-centre studies with different SGLT2i and in combination with various other agents. One of the most important limitations is the lack of RCTs comparing SGLT2i with a placebo. Although PCOS is a chronic disease, delaying active treatment in the comparator arm for a period of a few weeks or months would not possibly pose any risk or cause potential harm to the patients. The only placebo-controlled RCT lasted for just 2 weeks and was conducted using licogliflozin, an SGLT2i not commonly used globally. In addition, RCTs having active comparator arms were not powered for some of the important objective outcomes in PCOS. Moreover, most of the studies did not yield results on objective clinical measures, such as modified Ferriman–Gallwey scores for hirsutism and other clinical scores of androgenetic alopecia, acne or simple measures such as menstrual frequency. To observe meaningful differences in these clinical measures, it is important to extend the studies for a longer duration, spanning several months. There is an imminent need for focused and longer-duration placebo-controlled RCTs with SGLT2i in PCOS to establish these promising agents as recommended treatments in PCOS. One prior meta-analysis on four trials and a recently published meta-analysis on SGLT2i in PCOS have included the trials that are also included in this systematic review.^9,10^ We purposefully did not conduct a meta-analysis on this topic due to the small number of patients and the heterogeneity of the studies in terms of multiple different outcomes of interest, other active agents and comparator arms, and the wide range of follow-up periods ranging from 2 to 24 weeks.

## Conclusions

Overall, our results suggest a beneficial effect of SGLT2i on both hyperandrogenism and metabolic parameters in PCOS, with a positive impact on body composition; the beneficial effects were non-i nferior to metformin. Combining SGLT2i with metformin failed to demonstrate additional benefits on metabolic parameters but improved hyperandrogenism better than metformin alone. The combination of SGLT2i with GLP1RA showed promising results in improving metabolic parameters in PCOS and is an interesting therapy demanding further exploration. Dual SGLT1/2 inhibitors may have an edge over SGLT2i in reducing adrenal DHEAS production – an effect observed early after initiation and independent of weight loss. Larger studies, especially randomized trials comparing SGLT2 inhibitors, metformin, their combinations and placebo, are the need of the hour to better understand their metabolic, hormonal, glycaemic and clinical outcomes.
